# Climate Change and Human Health in China

**DOI:** 10.1289/ehp.1003354

**Published:** 2011-02

**Authors:** Haidong Kan

**Affiliations:** Fudan University, Shanghai, China, E-mail: haidongkan@gmail.com

There is near unanimous scientific consensus that the world’s climate is changing and most of the warming is attributable to human activities. Over the past few decades, economic expansion of China, the largest developing country, has been one of the strongest in world history. Such an economic expansion, however, is largely driven by fossil fuels, which leads to dramatic increases in emissions of greenhouse gases (GHGs). Although emissions per person in China are at the global average, China surpassed the United States as the country emitting the most carbon dioxide (CO_2_) in 2007 (International Energy Agency 2009). China is also a large emitter of methane and black carbon, the other two major contributors to global warming.

As in other parts of the world, China has experienced noticeable changes in climate over the past 100 years (National Development and Reform Commission 2007). The annual average air temperature has risen by 0.5–0.8°C, which is slightly higher than the average global temperature increase, and most of these changes have been observed over the past 50 years. Regional distribution of temperature changes shows that the warming trend was more significant in western, eastern, and northern China than in southern China. Seasonal distribution of the temperature changes shows that the most significant temperature increase occurred in winter. The trend of climate warming in China is projected to intensify in the future.

The Chinese government has paid great attention to climate change, but so far, there has been limited focus on climate-related the health impacts. Evidence is mounting that climate change has already affected human health directly and indirectly in China, including mortality from extreme weather events, changes in air and water quality, and changes in the ecology of infectious diseases (Zhang et al. 2010).

The observed relationship between temperature and daily mortality among Chinese residents has been generally J- or U-shaped, with mortality risk decreasing from the lowest temperature to an inflection point and then increasing with higher temperature (Kan et al. 2007). Heat waves and other extreme weather conditions have been associated with increased death risk in large Chinese cities, such as Beijing and Shanghai. Elevated mortality during temperature extremes has been attributed mainly to cardiovascular and respiratory diseases, especially among the elderly. However, little information is available in China on possible modifiers of the health impact of thermal extremes, such as preexisting health status and population demographics.

Improvement of living conditions resulting from rapid economic development in China can reduce heat wave–related heath impacts. In Shanghai, for example, heat waves in 1998 and 2003 both led to increased mortality. Although the heat waves in these 2 years were similar in meteorological characteristics, elevated mortality was much less pronounced during the 2003 event, suggesting that adaptations to climate change (e.g., increased use of air conditioners, larger living spaces, increased urban green space, higher levels of heat awareness, implementation of a heat warning system issued by local meteorological stations) can reduce health risks imposed on Chinese residents (Tan et al. 2007).

The health impacts of high temperatures and severe air pollution may interact and are thus worthy of attention, because outdoor air pollution is one of the major environmental challenges for public health in Chinese cities. In Wuhan, an “oven” city because of its hot summers, high temperatures enhanced the effect of particulate matter ≤ 10 μm (PM_10_) on cardiopulmonary mortality, even though PM_10_ concentrations were lower on days with extremely high temperatures than on days with normal and low temperatures (Qian et al. 2008). Also, levels of some secondary air pollutants, such as ozone, are affected by temperature and tend to be higher on hot days. Epidemiological evidence from Chinese cities has indicated significant risks of ozone associated with increasing temperatures (Zhang et al. 2006).

Climate change may lead to a wide range of extreme weather events in China, including typhoons, floods, blizzards, windstorms, drought, and landslides. The direct and indirect health effects due to these extreme events are important but are difficult to assess at present.

Climate change can also affect climate-sensitive infectious diseases carried by animal hosts or vectors; in China, these include schistosomiasis, Japanese encephalitis, Dengue fever, malaria, and *Angiostrongylus cantonensis* infection. For example, schistosomiasis is one of the vector-borne diseases that received little attention for many years, but the impact of climate change on its transmission has recently attracted attention and debate. In a recent study, Zhou et al. (2010) found that as winter temperatures continue to rise due to global warming, *Oncomelania hupensis* (the intermediate host of *Schistosoma japonicum* in China) may increase its range, thereby spreading schistosomiasis to the northern part of China.

China is striving to quadruple its gross domestic product (GDP) of the year 2000 by 2020, and consequently will face even more serious challenges in controlling emission of GHGs. The anthropogenic contribution to GHGs is largely caused by the same activity that causes air pollution, so if China acts to reduce the combustion of fossil fuels and the resultant air pollution, it will reap not only the health benefits associated with improvement of air quality but also the climate change benefits. Therefore, these air pollution–related health benefits might be a strong inducement for the Chinese government to act to combat climate change.

In short, there is sufficient evidence showing that climate change has posed health risks to Chinese populations. Future research will need to provide methods of adapting to climate change and evaluate the implementation of adaptation measures; improve characterization of climate–health relationships (particularly at regional levels); identify thresholds and particularly vulnerable groups; and collect and enhance long-term surveillance data on health issues of potential concern (including morbidity due to temperature extremes, vector-borne diseases, air quality, pollen and mold counts, food-borne and waterborne diseases, and mental health impacts from extreme weather events). Finally, GHG emissions need to be controlled. Concern about the health impacts of climate change can encourage the Chinese government to take appropriate action.

## Figures and Tables

**Figure:**
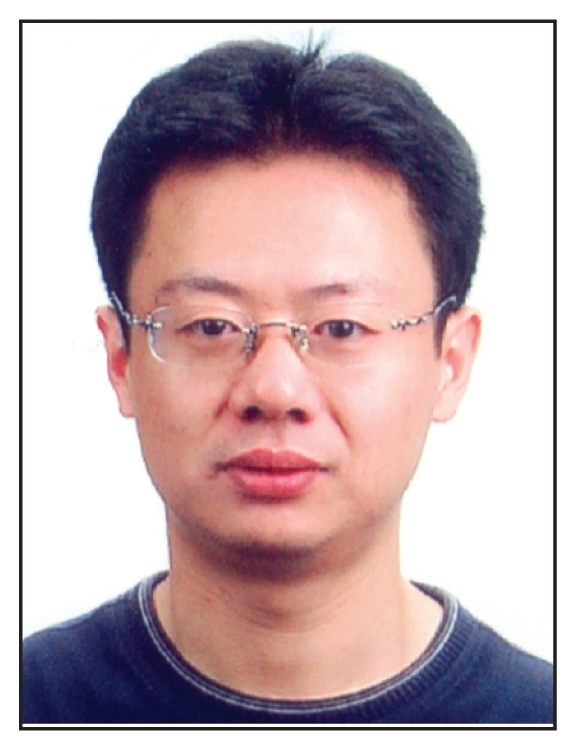
Haidong Kan
